# The role of α-synuclein prion strains in Parkinson’s disease and multiple system atrophy

**DOI:** 10.1371/journal.ppat.1011920

**Published:** 2024-01-25

**Authors:** Chase R. Khedmatgozar, Sara A. M. Holec, Amanda L. Woerman

**Affiliations:** Department of Microbiology, Immunology, and Pathology, Prion Research Center, Colorado State University, Fort Collins, Colorado, United States of America; National Institutes of Health, UNITED STATES

In 1817, James Parkinson published *An Essay on the Shaking Palsy*, which identified 6 patients with a disorder defined by a gradual weakening of muscles, stooped posture, abnormal gait, resting tremors, impaired dexterity, sleep disruption, and difficulty controlling bowel movements [1]. Almost 100 years later, Frederic Lewy described the presence of insoluble protein inclusions in neurons in patients with the same clinical presentation, which are now referred to as Lewy bodies (LBs) and are the defining pathological hallmark of Parkinson’s disease (PD) [2]. Just 12 years prior, Dejerine and Thomas described a disorder they named olivopontocerebellar atrophy, which was characterized by impaired dexterity, abnormal speech, facial paralysis, and difficulties walking and controlling urinary movements [3]. Similar disorders, including striatonigral degeneration and Shy-Drager syndrome [4,5], were later recognized to be clinical manifestations of the same disorder, and the all-encompassing term “multiple system atrophy” (MSA) was adopted [6]. By 1989, Papp and colleagues identified the presence of aggregated protein in oligodendrocytes, later named glial cytoplasmic inclusions (GCIs), in brains from patients with olivopontocerebellar atrophy, striatonigral degeneration, and Shy-Drager syndrome, concretely unifying the 3 diseases as MSA under this defining neuropathological feature [7]. While PD and MSA were largely thought to be 2 unrelated neurodegenerative disorders prior to the 1990s, several monumental discoveries in the following decade led to the recognition that the disorders are linked by a shared pathogenic mechanism.

In 1997, 2 convergent studies implicated the protein α-synuclein in PD. In the first, genetic analysis of samples collected from the Contursi kindred in Italy, which has a high prevalence of early-onset PD, led to the discovery of a familial mutation in *SNCA*, the gene encoding α-synuclein [8]. Simultaneously, Spillantini and colleagues used immunohistochemical labeling of fixed brain sections from PD patient brains to show that LBs are primarily comprised of aggregated α-synuclein [9]. Notably, while the genetics studies pointed to the importance of the A53T mutation in α-synuclein as driving an autosomal dominant form of disease, the immunolabeling studies revealed that α-synuclein mutations are not required for the formation of LBs or PD. Less than one year later, both Spillantini and colleagues and Wakabayashi and colleagues reported that the GCIs in MSA patient samples are also primarily comprised of α-synuclein, linking the 2 diseases as synucleinopathies [10,11]. However, unlike in PD, in which multiple point mutations give rise to disease, no *SNCA* mutations have been identified in MSA patients.

## Distinct α-synuclein strains give rise to PD and MSA

**Fig 1:**
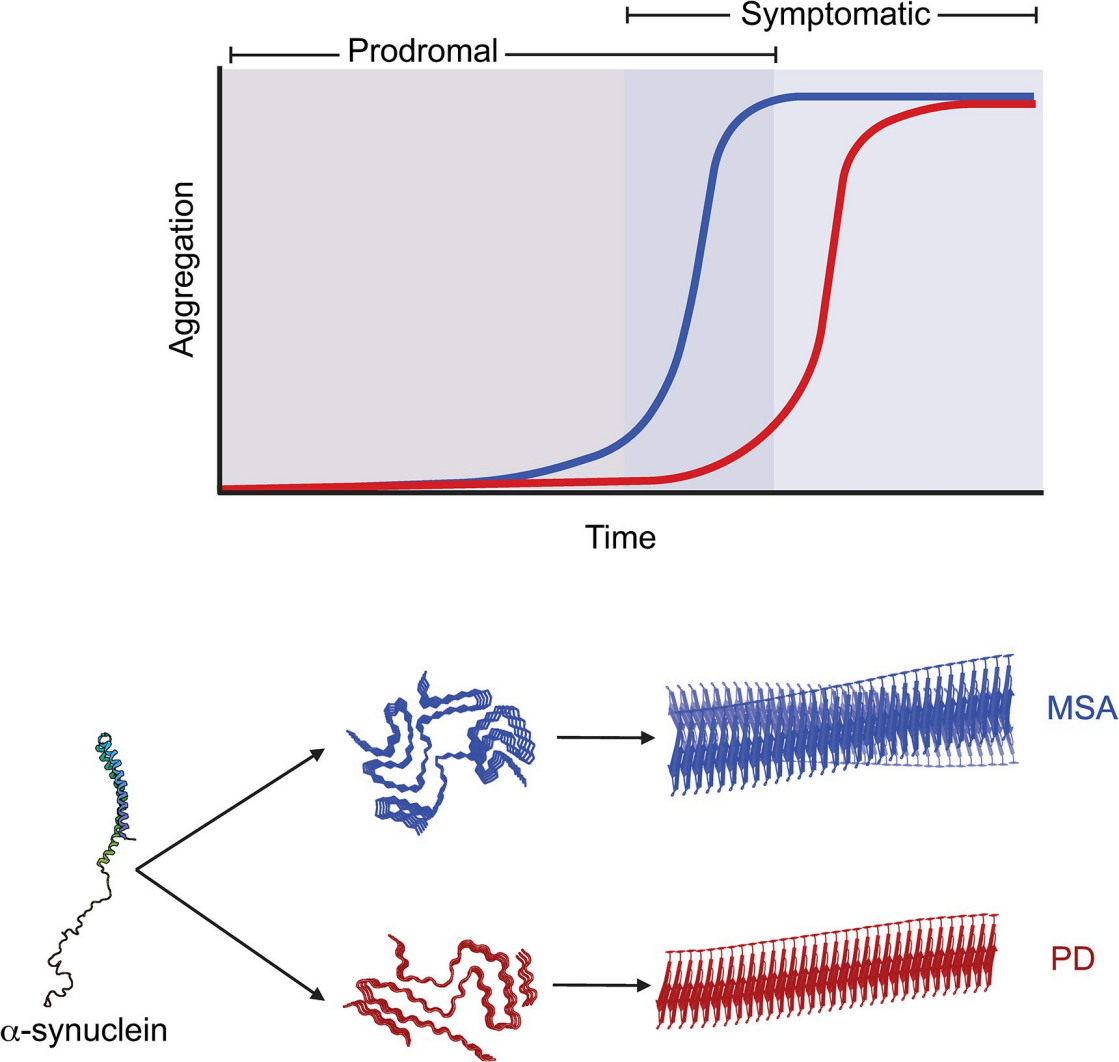
The rate of disease progression is strain specific. The time required for a patient to become symptomatic is determined by the energy required for each α-synuclein monomer to adopt a particular misfolded conformation (MSA shown in blue and PD shown in red). Notably, spreading of misfolded α-synuclein occurs during both the prodromal and symptomatic periods of disease. Cryo-electron microscopy studies have shown that α-synuclein misfolds into 2 heterotypic protofilaments in MSA, which grow to form a distinct twisting filament (PDB ID: 6XYO). By comparison, the single protofilament of PD leads to the formation of a filament with minimal twist (PDB ID: 8A9L). Figure was created with BioRender.com.

The ability of a single protein to cause multiple diseases is a unique biological phenomenon. The initial studies investigating this observation focused on the role of the prion protein (PrP) in a group of fatal neurodegenerative disorders afflicting humans and other mammalian species. In these diseases, the cellular form of PrP (PrP^C^) misfolds into an aberrant conformation (PrP^Sc^), which can induce additional misfolding by imprinting itself onto native PrP^C^ [12]. This cascade of PrP^Sc^ misfolding, or the prion mechanism of disease, enables prion replication to spread throughout the brain, causing progressive neurodegeneration. An early criticism of the prion hypothesis was the inability to explain how PrP^Sc^ could cause a variety of diseases, both between and across species, despite the absence of a nucleic acid, including scrapie, bovine spongiform encephalopathy, Creutzfeldt-Jakob disease, and fatal familial insomnia. What ultimately emerged was the *strain hypothesis*, which proposes that PrP^Sc^ misfolds into multiple distinct conformations, or strains, that give rise to unique biophysical and biological properties that ultimately manifest as individual clinical phenotypes (Fig 1) [13]. Following the discovery that α-synuclein is the main neuropathological component in both PD and MSA, research over the last 30 years has focused on the ability of α-synuclein to misfold into a prion that adopts distinct disease-causing conformations.

### Structural differences in α-synuclein lead to distinct strain biologies

**Fig 2:**
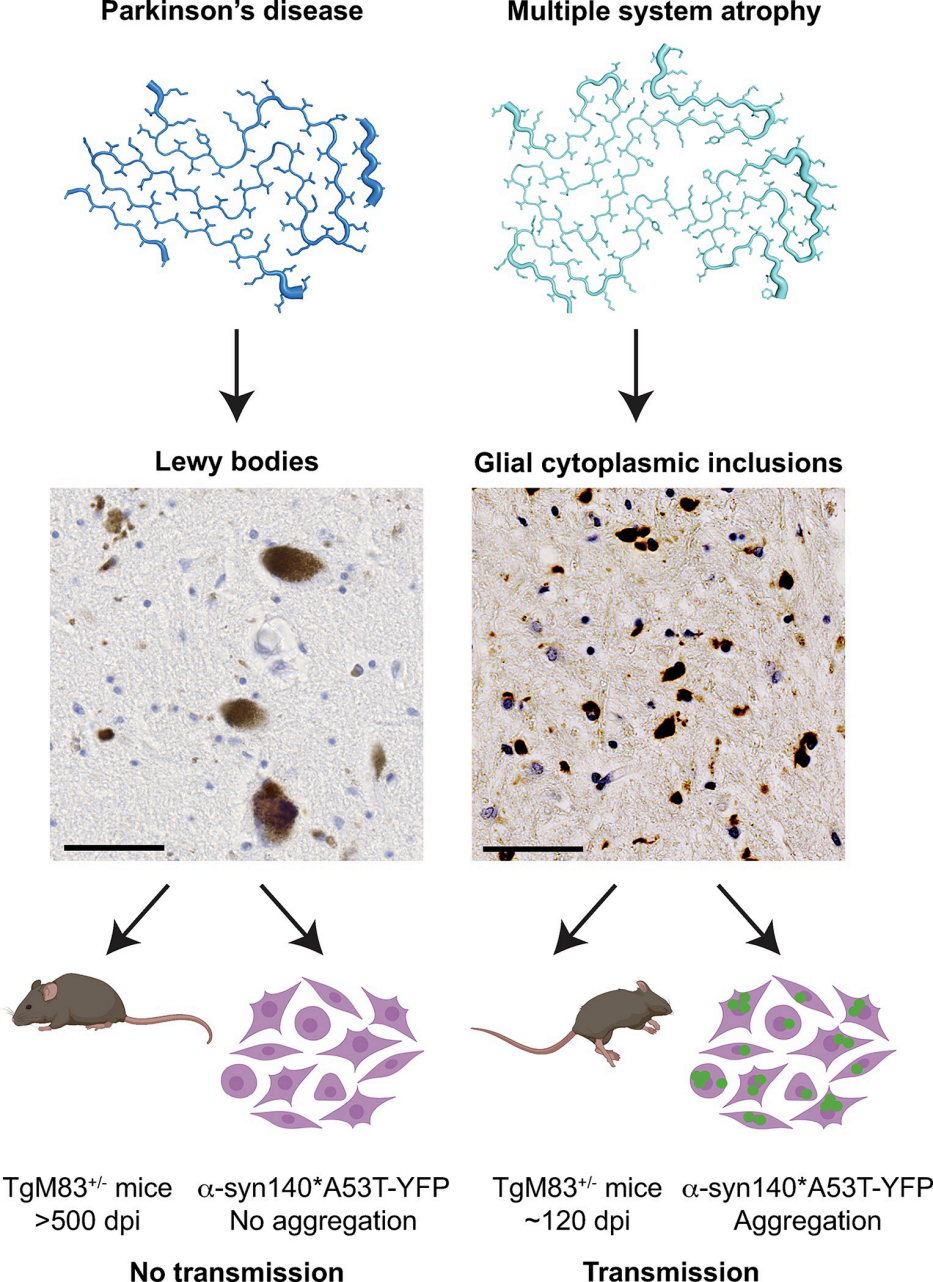
Distinct α-synuclein strains exhibit unique biological activities. (Top) Misfolded α-synuclein fibrils isolated from PD (left) and MSA (right) patient samples adopt distinct conformations. PD fibrils (PDB ID: 8A9L) are shown in blue and MSA fibrils (PDB ID: 6XYO) are shown in teal. (Middle) Each α-synuclein strain gives rise to a distinct neuropathological hallmark in the brain. PD patients develop neuronal LBs (left), whereas MSA patients develop glial cytoplasmic inclusions in oligodendrocytes (right). Scale bar, 50 μm. (Bottom) The 2 α-synuclein strains have opposite biological effects when transmitted to 2 research models. TgM83^+/−^ mice expressing human α-synuclein with the A53T mutation remain asymptomatic >500 dpi when inoculated intracranially with PD patient samples. TgM83^+/−^ mice inoculated with MSA patient samples develop neurological disease in approximately 120 dpi. Similarly, α-synuclein prions isolated from PD patient samples are unable to replicate in HEK293T cells expressing YFP-tagged α-synuclein with the A53T mutation; however, α-synuclein prions isolated from MSA patient samples easily replicate and induce YFP-positive puncta in the cells. Figure was created with BioRender.com. dpi, days postinoculation; LB, Lewy body; MSA, multiple system atrophy; PD, Parkinson’s disease; YFP, yellow fluorescent protein.

To understand the structural differences in α-synuclein between PD and MSA patients, recent advances in cryogenic-electron microscopy (cryo-EM) have enabled the resolution of α-synuclein fibrils isolated from patient samples [14,15]. These studies have shown notable differences in α-synuclein fibril structure, including the discovery that the PD filament contains a single protofilament, whereas the MSA fibril contains 2 asymmetric protofilaments (Figs 1 and 2). This difference likely has major impacts on the formation and stability of the 2 types of aggregates. Additional differences include the presence of a salt bridge between residues E35 and K80 in the PD structure, whereas the stabilizing salt bridge is between residues E46 and K80 in the MSA structure. Recent findings have shown that this structural difference is responsible for distinct biological properties between the PD and MSA strain in both in vitro and in vivo models [16,17]. Finally, differences in N-terminal contributions are also likely to alter fibril kinetics and stability. Despite these variations, an important shared feature between the 2 fibrils is the presence of several similarly sized nonproteinaceous densities, which are likely cofactors that contribute to the structural stability of each conformation. These exciting findings clearly demonstrate that distinct α-synuclein conformations underlie important biochemical and biological differences between the 2 disorders.

### Strain differences give rise to unique biochemical properties

A defining characteristic of prions is their highly stable nature, even in the presence of stringent denaturants and enzymes. This is thought to be due to the large number of hydrogen bonds that form between the carbon backbones of misfolded proteins in a fibril. Notably, PrP^Sc^ strains have traditionally been defined based on differences in their resistance to denaturation by chemicals [e.g., guanidine hydrochloride (GdnHCl)] or enzymes [e.g., proteinase K (PK)], which are likely determined by a mixture of strain-specific factors including the number of residues involved in PrP^Sc^ templating and accessibility of the fibril core. Several studies have used similar methods to interrogate α-synuclein strain properties across synucleinopathy patient samples. For example, GdnHCl denaturation assays showed that α-synuclein from PD patient samples is more stable than α-synuclein from MSA samples [18]. Similarly, α-synuclein strains are differentially susceptible to PK digestion and sarkosyl denaturation [19,20].

### α-Synuclein prions spread from cell to cell

Prions are also defined by their ability to self-propagate and cause progressive disease. In 2003, Braak and colleagues described a specific pattern by which pathogenic α-synuclein moves through the body from cell to cell in a subset of PD patients [21]. This hypothesis was bolstered in 2008, when fetal grafts implanted into the substantia nigra of PD patients later developed Lewy pathology, demonstrating cell-to-cell spread of α-synuclein prions [22,23]. Accounting for differences in clinical presentation, α-synuclein pathology impacts specific brain regions in PD and MSA. For example, while PD patients can develop pathology in the olfactory bulb, cortices, midbrain, and brainstem, MSA patients develop pathology in the basal ganglia, amygdala, hypothalamus, and brainstem [24,25]. While it is well known that prion strains target unique subsets of brain regions, the mechanism underlying this phenomenon is not known.

### Propagation of α-synuclein prions in research models

Of the substantive animal models used to investigate synucleinopathies, the most frequently used is the TgM83 mouse model, which expresses human α-synuclein with the A53T mutation [26]. Homozygous mice develop spontaneous disease defined by motor impairment after 1 year; however, hemizygous mice remain asymptomatic beyond 500 days [27]. Watts and colleagues first demonstrated that MSA transmits neurological disease and α-synuclein neuropathology to TgM83^+/−^ mice following intracranial inoculation of 2 MSA patient samples [27]. Subsequent studies passaging these initial brain homogenates demonstrated that clinical phenotypes and pathological lesions are maintained upon serial passage [28]. Moreover, TgM83^+/−^ mice also develop neurological disease following intramuscular (tongue or hind leg), intraperitoneal, or intravenous exposure [19,29], though transmission efficiency was low for intralingual injections.

Finally, cell culture models have also been used to investigate differences in α-synuclein strain biology, with the notable ability to predict in vivo outcomes [17,28,30,31]. For example, PD patient samples do not transmit disease to TgM83^+/−^ mice, nor do they infect HEK293T cells expressing YFP-tagged α-synuclein. This lack of transmissibility may be due to differences in the strain replication rate, the stability of PD versus MSA fibrils, the templating interface, or cofactor availability in these models. Additionally, as noted previously, MSA prions are stabilized by a salt bridge between residues E46 and K80. Cell-based studies showed that the presence of the PD-causing E46K mutation inhibits replication of MSA prions in vitro [16], which was subsequently confirmed by in vivo experiments finding transgenic mice expressing the E46K mutation are protected from MSA transmission [17].

## Conclusions

Mounting data, including structural, biochemical, and biological findings, demonstrate that 2 distinct α-synuclein prion strains underlie disease pathogenesis in MSA and PD patients. The tools developed to identify and investigate the consequences of these strain differences have also shown that the misfolding and accumulation of α-synuclein prions occurs long before symptoms arise. As research on synucleinopathies moves forward, it is imperative that the field draws from recent breakthroughs to focus on incorporating strain biology into the development of quantitative biomarkers, diagnostics, and disease-modifying therapeutics. In doing so, we have an opportunity to generate meaningful translational research with profound impacts on patients, their family members, and their caregivers.
